# Bayesian Input Design for Linear Dynamical Model Discrimination

**DOI:** 10.3390/e21040351

**Published:** 2019-03-30

**Authors:** Piotr Bania

**Affiliations:** Department of Automatic Control and Robotics, AGH University of Science and Technology, Al. A. Mickiewicza 30, 30-059 Krakow, Poland; pba@agh.edu.pl; Tel.: +48-12-617-28-34

**Keywords:** bayesian experimental design, model discrimination, information, entropy

## Abstract

A Bayesian design of the input signal for linear dynamical model discrimination has been proposed. The discrimination task is formulated as an estimation problem, where the estimated parameter indexes particular models. As the mutual information between the parameter and model output is difficult to calculate, its lower bound has been used as a utility function. The lower bound is then maximized under the signal energy constraint. Selection between two models and the small energy limit are analyzed first. The solution of these tasks is given by the eigenvector of a certain Hermitian matrix. Next, the large energy limit is discussed. It is proved that almost all (in the sense of the Lebesgue measure) high energy signals generate the maximum available information, provided that the impulse responses of the models are different. The first illustrative example shows that the optimal signal can significantly reduce error probability, compared to the commonly-used step or square signals. In the second example, Bayesian design is compared with classical average D-optimal design. It is shown that the Bayesian design is superior to D-optimal design, at least in this example. Some extensions of the method beyond linear and Gaussian models are briefly discussed.

## 1. Introduction

Discrimination of various dynamical models of the same process has a wide area of applications, especially in multiple-model fault detection and isolation [1,2,3,4] and, in many other estimation and control problems [5,6,7], it is necessary to choose the most likely dynamical model from a finite set. The discrimination task can be formulated as an estimation problem, where the estimated parameter θ indexes particular models. The problem can also be considered as a finite-dimensional approximation of more general identification tasks [8]. As the error probability or variance of the estimator of θ usually depends on the input signal, it is important to select a signal that minimizes error probability or maximizes a utility function that encodes the purpose of the experiment. Selection of an input signal that maximizes a utility function is strongly related to optimal experimental design [9].

Experimental design methods can be divided into classical and Bayesian. The classical methods, also called optimal experimental design, typically use various functionals of the Fisher information matrix as a utility function. These methods are widely described in the literature and work well if the model is linear in its parameters (see [8,10,11,12] and the review article [9]). Unfortunately, in typical identification tasks, the solution of model equation and the covariance depends non-linearly on θ, even if the model equation is linear. This implies that the information matrix and the utility function depend on the parameter θ to be estimated. Therefore, only locally-optimal design can be obtained [13]. To obtain more robust methods, an averaging over the prior parameter distribution or minimax design [8,14] (Section 6.1) are commonly used, but these methods are not fully Bayesian.

Bayesian optimal design uses the utility function, a functional of the posterior distribution (see [15,16] and the review articles [13,17,18]). The most commonly used utility functions are mutual information between parameters and model output, Kullback-Leibler divergence between the prior and posterior distributions, and the determinant of the posterior covariance matrix [13,16]. In contrast to classical methods, in Bayesian design the utility function does not depend on the parameters to be estimated. Hence, the method can cope with non-linear problems. The utility function, which is suitable for model discrimination, is the error probability of the MAP estimator of θ [19]. Such a utility function is generally difficult to calculate (see [19]), but the result of Feder & Merhav [20] implies that the error probability of the MAP estimator is upper-bounded by some decreasing function of mutual information between θ and the output of the system. Hence, the maximization of mutual information creates the possibility of reducing the error probability, provided that appropriate estimator is used. However, the most serious problem that inhibits the development of this idea is great computational complexity in calculating the mutual information.

The main contribution of this article is a fully-Bayesian (in the terminology of [13]) method for finding an input signal that maximizes the mutual information between θ and the system output. Maximization of information or, equivalently, maximization of the output entropy has been proposed by many authors (see, e.g., [13,15,17,18,21,22,23]), but the mutual information is very hard to compute and the problem is often intractable. To overcome this serious difficulty, instead of mutual information the lower bound, given by Kolchinsky & Tracey [24], has been used. This is a pairwise-distance based entropy estimator and it it useful here, since it is differentiable, tight, and asymptotically reaches the maximum possible information (see [24] (Sections 3.2, 4, 6)). Maximization of such a lower bound, under the signal energy (i.e., the square of the signal norm) constraints, is much simpler, gives satisfactory solutions, and allows for practical implementation of the idea of maximizing information. This is illustrated with examples. Moreover, it is shown that, for certain cases, this problem reduces to a solution of a certain eigenproblem.

The article is organized as follows. In Section 2, the estimation task is formulated and the upper bound of the error probability and the lower bound of the mutual information are given. In Section 2.1, a selection between two models is discussed and an exact solution is given. Design of input signals with small energy, which is required in some applications, is described in Section 2.2. In Section 2.3, the large energy limit is discussed. An application to linear dynamical systems with unknown parameters is given in Section 3. An example of finding the most likely model among three stochastic models with different structures is given in Section 4. Comparison with classical D-optimal design is performed in Section 5. The article ends with conclusions and references.

## 2. Maximization of Mutual Information between the System Output and Parameter

Let us consider a family of linear models

(1) $$Y=F_{\theta }U+Z,$$

where θ∈1,2,...,r, $Y,Z\in R^{n_{Y}}$, and $U\in R^{n_{U}}$. The matrices $F_{\theta }$ are bounded. The parameter θ is unknown. The prior distribution of θ is given by

(2) $$P\left(\theta =i\right)=p_{0,i},i=1,...,r.$$

The random variable *Z* is conditionally normal (i.e., $p\left(Z|\theta \right)=N\left(Z,0,S_{\theta }\right)$), where the covariance matrices $S_{\theta }$ are given a priori and $S_{\theta }>0$, for all θ. The variable *U* is called the input signal. In all formulas below, the input signal *U* is a deterministic variable. The set of admissible signals is given by

(3) $$S_{\varrho }=\left\{U\in R^{n_{U}};U^{T}U⩽\varrho \right\}.$$

Under these assumptions, and after applying Bayes rule:

(4) $$\begin{array}{c} p\left(Y|U\right)=\sum_{\theta =1}^{r}p_{0,\theta }N\left(Y,F_{\theta }U,S_{\theta }\right), \end{array}$$

(5) $$\begin{array}{c} p\left(Y|\theta ,U\right)=N\left(Y,F_{\theta }U,S_{\theta }\right), \end{array}$$

(6) $$\begin{array}{c} p\left(\theta |Y,U\right)=\frac{p_{0,\theta }N\left(Y,F_{\theta }U,S_{\theta }\right)}{\sum_{j=1}^{r}p_{0,j}N\left(Y,F_{j}U,S_{j}\right)}. \end{array}$$

The entropies of *Y* and θ and the conditional entropies are defined as

(7) $$\begin{array}{c} H\left(\theta \right)=-\sum_{\theta =1}^{r}p_{0,\theta }\ln p_{0,\theta }, \end{array}$$

(8) $$\begin{array}{c} H\left(\theta |Y,U\right)=-\int p\left(Y|U\right)\left(\sum_{\theta =1}^{r}p\left(\theta |Y,U\right)\ln p\left(\theta |Y,U\right)\right)dY, \end{array}$$

(9) $$\begin{array}{c} H(Y|U)=-\int p(Y|U)\ln p(Y|U)dY, \end{array}$$

(10) $$\begin{array}{c} H\left(Y|\theta \right)=\frac{1}{2}\sum_{\theta =1}^{r}p_{0,\theta }\ln\left({\left(2\pi e\right)}^{n_{y}}\left|S_{\theta }\right|\right). \end{array}$$

The mutual information between θ and *Y* is defined as (see [25] (pp. 19, 250))

I(Y;θ|U)=H(θ|U)−H(θ|Y,U)=H(Y|U)−H(Y|θ,U).

As H(θ|U)=H(θ) and H(Y|θ,U)=H(Y|θ), then I(Y;θ|U) is given by

(11) I(Y;θ|U)=H(θ)−H(θ|Y,U)=H(Y|U)−H(Y|θ).

The MAP estimator of θ is defined as

$$\hat{\theta }\left(Y,U\right)=\arg\max_{\theta \in \{1,...,r\}}p\left(\theta |Y,U\right).$$

The error probability of $\hat{\theta }$ is given by (see [20])

$$P_{e}\left(U\right)=1-\int \left(\max_{\theta \in \{1,...,r\}}p\left(\theta |Y,U\right)\right)p\left(Y|U\right)dY.$$

It follows from Fano’s inequality ([25] (p. 38)), that $P_{e}$ is lower bounded by an increasing function in H(θ|Y,U). Feder & Merhav [20] proved that $2P_{e}\left(U\right)⩽H\left(\theta |Y,U\right)\log_{2}e$. As H(θ|Y,U)=H(θ)−I(Y;θ|U) and H(θ) does not depend on *U*, then the maximization of I(Y;θ|U) creates the possibility of reducing $P_{e}$, and the optimal signal is given by

(12) $$U^{*}\left(\varrho \right)=\arg\max_{U\in S_{\varrho }}I\left(Y;\theta |U\right).$$

To overcome the problems associated with the calculation of I(Y;θ|U), we will use its lower bound.

**Lemma 1.**  *(Information bounds). For all $U\in R^{n_{U}}$,*

(13) $$I_{l}\left(U\right)⩽I\left(Y;\theta |U\right)⩽H\left(\theta \right),$$

*where*

(14) $$\begin{array}{c} I_{l}\left(U\right)=-\sum_{i=1}^{r}p_{0,i}\ln\left(\sum_{j=1}^{r}p_{0,j}e^{-D_{i,j}\left(U\right)}\right), \end{array}$$

(15) $$\begin{array}{c} D_{i,j}\left(U\right)=\frac{1}{4}U^{T}Q_{i,j}U+\frac{1}{2}\ln|\frac{1}{2}\left(S_{i}+S_{j}\right)|-\frac{1}{4}\ln|S_{i}\left|\right|S_{j}|,\;\mathrm{and} \end{array}$$

(16) $$\begin{array}{c} Q_{i,j}={\left(F_{i}-F_{j}\right)}^{T}{\left(S_{i}+S_{j}\right)}^{-1}\left(F_{i}-F_{j}\right). \end{array}$$

**Proof.**  According to (4), p(Y|U) is finite Gaussian mixture. For such mixtures, the information bounds are known. A detailed proof, based on Chernoff α-divergence, is given in [24] (Section 4). □

**Lemma** **2.** *Let $\hat{\theta }\left(Y,U\right)=\arg\max_{\theta \in \{1,...,r\}}p\left(\theta |Y,U\right)$ be the MAP estimator of θ, and let $P_{e}\left(U\right)$ denote its error probability. There exists a continuous, increasing, and concave function $f:\left[0,H\left(\theta \right)\right]\to \left[0,1-r^{-1}\right]$, such that*

(17) $$P_{e}\left(U\right)⩽f\left(H\left(\theta \right)-I_{l}\left(U\right)\right)⩽\frac{1}{2}\left(H\left(\theta \right)-I_{l}\left(U\right)\right)\log_{2}e.$$

**Proof.**  Feder & Merhav [20] (see Theorem 1 and Equation (14)) proved that there exists an increasing, continuous, and convex function $\phi :\left[0,1-r^{-1}\right]\to \left[0,H\left(\theta \right)\log_{2}e\right]$, such that

(18) $$2P_{e}\left(U\right)⩽\phi \left(P_{e}\left(U\right)\right)⩽H\left(\theta |Y,U\right)\log_{2}e.$$

As H(θ|Y,U)=H(θ)−I(Y;θ|U) and $I_{l}\left(U\right)⩽I\left(Y;\theta |U\right)$, then $\phi \left(P_{e}\left(U\right)\right)⩽\left(H\left(\theta \right)-I_{l}\left(U\right)\right)\log_{2}e$. The function $g=\phi^{-1}$ is increasing, continuous, concave, and it follows from (18) that 2g(η)⩽η. Hence, $P_{e}\left(U\right)⩽\phi^{-1}\left(\left(H\left(\theta \right)-I_{l}\left(U\right)\right)\log_{2}e\right)=g\left(\left(H\left(\theta \right)-I_{l}\left(U\right)\right)\log_{2}e\right)⩽\frac{1}{2}\left(H\left(\theta \right)-I_{l}\left(U\right)\right)\log_{2}e$. Taking $f\left(\eta \right)=g\left(\eta \log_{2}e\right)$ we obtain the result. □

Now, the approximate solution of (12) is given by

(19) $$U^{*}\left(\varrho \right)=\arg\max_{U\in S_{\varrho }}I_{l}\left(U\right).$$

As $I_{l}$ is smooth and $S_{\varrho }$ is compact, (19) is well-defined.

### 2.1. Selection between Two Models

Suppose that θ takes only two values, 1 and 2, with prior probabilities $p_{0,1}$ and $p_{0,2}=1-p_{0,1}$, respectively. It’s easy to check, by direct calculation, that

(20) $$e^{-I_{l}\left(U\right)}={\left(p_{0,1}+p_{0,2}e^{-D_{1,2}\left(U\right)}\right)}^{p_{0,1}}{\left(p_{0,1}e^{-D_{1,2}\left(U\right)}+p_{0,2}\right)}^{p_{0,2}}.$$

Equation (20) implies that the maximization of $I_{l}$ is equivalent to the maximization of $D_{1,2}$. On the basis of (15), we have the following optimization: task

(21) $$\max_{U^{T}U⩽\varrho }U^{T}Q_{1,2}U.$$

The solution of (21) is the eigenvector of $Q_{1,2}$ corresponding to its largest eigenvalue; that is,

(22) $$Q_{1,2}U^{*}=\lambda_{max}\left(Q_{1,2}\right)U^{*},||U^{*}{\left|\right|}^{2}=\varrho .$$

### 2.2. Small Energy Limit

In many practical applications, the energy of an excitation signal must be small. The second order Taylor expansion of (14) gives

(23) $$I_{l}\left(U\right)=\frac{1}{4}U^{T}QU-\sum_{i=1}^{r}p_{0,i}\ln\sum_{j=1}^{r}\alpha_{i,j}+{o(||U||}^{2}),$$

where

(24) $$Q=\sum_{i=1}^{r}p_{0,i}\left(\frac{\sum_{j=1}^{r}\alpha_{i,j}Q_{i,j}}{\sum_{j=1}^{r}\alpha_{i,j}}\right),\;\mathrm{and}$$

(25) $$\alpha_{i,j}=p_{0,j}e^{-D_{i,j}\left(0\right)}.$$

If the value of ϱ (see (3)) is small, then the last term in (23) can be omitted. As the second term does not depend on *U*, we have the following optimization task:

(26) $$\max_{U^{T}U⩽\varrho }U^{T}QU.$$

The solution of (26) is the eigenvector of *Q* corresponding to its largest eigenvalue.

### 2.3. Large Energy Limit

We will investigate asymptotic behaviour of I(Y;θ|U) when ||U||→∞. On the basis of Lemma 1, the condition

(27) $$\min_{i\neq j}U^{T}Q_{i,j}U>0$$

guarantees that $\lim_{\varrho \to \infty }I\left(Y;\theta |\varrho U\right)=H\left(\theta \right)$. It is also possible that $\lim_{\varrho \to \infty }I\left(Y;\theta |\varrho U\right)<H\left(\theta \right)$, for some *U*. Such signals are weakly informative and they cannot generate the maximum information, even if their amplitude tends to infinity. Let $S_{1}$ denote the unit ball in $R^{n_{U}}$ and let μ be the Lebesgue measure on $S_{1}$. The set of weakly informative signals is defined as

(28) $$\Omega =\{U\in S_{1}:\lim_{\varrho \to \infty }I\left(Y;\theta |\varrho U\right)<H\left(\theta \right)\}.$$

**Theorem** **1.** *μ(Ω)=0 if and only if $F_{i}\neq F_{j}$, for all i≠j.*

**Proof.**  ⇐: On the basis of Lemma 1 and (28), $\Omega =\cup_{i\neq j}\Omega_{i,j}$, where $\Omega_{i,j}=\left\{\xi \in S_{1}:\xi^{T}Q_{i,j}\xi =0\right\}$. Since $S_{i}+S_{j}$ is positive-definite and $F_{i}\neq F_{j}$ then, on the basis of (16), the matrix $Q_{i,j}$ has at least one positive eigenvalue. Hence, $\mu (\Omega_{i,j})=0$ and $\mu \left(\Omega \right)=\sum_{i\neq j}\mu \left(\Omega_{i,j}\right)=0$. ⇒: The condition μ(Ω)=0 implies that $\mu (\Omega_{i,j})=0$. Hence, $Q_{i,j}$ has at least one positive eigenvalue, which is possible only if $F_{i}\neq F_{j}$. □

As a conclusion, we have the following result.

**Theorem** **2.** *Let $\hat{\theta }\left(Y,U\right)=\arg\max_{\theta \in \{1,...,r\}}p\left(\theta |Y,U\right)$, be the MAP estimator of θ and let $P_{e}\left(U\right)$ denote its error probability. If $F_{i}\neq F_{j}$ for all i≠j, then, for any ϵ>0, there exists a number ϱ>0 and a signal $U\in S_{\varrho }$, such that $P_{e}\left(U\right)<\epsilon$.*

**Proof.**  By the assumption, and from Theorem 1, the set $S_{1}\setminus \Omega$ is non-empty. If $U\in S_{1}\setminus \Omega$, then $\min_{i\neq j}U^{T}Q_{i,j}U>0$ and, from Lemma 1, we get $\lim_{\varrho \to \infty }I_{l}\left(\varrho U\right)=\lim_{\varrho \to \infty }I\left(Y;\theta |\varrho U\right)=H\left(\theta \right)$. Now, Lemma 2 implies that $2P_{e}\left(U\right)⩽\left(H\left(\theta \right)-I_{l}\left(\varrho U\right)\right)\log_{2}e<\epsilon$, for sufficiently large ϱ. □

## 3. Application to Linear Dynamical Systems

Consider, now, the family of linear systems

(29) $$\begin{array}{c} x_{k+1}=A_{\theta }x_{k}+B_{\theta }u_{k}+G_{\theta }w_{k},k=0,1,2,...,N-1, \end{array}$$

(30) $$\begin{array}{c} y_{k}=C_{\theta }x_{k}+D_{\theta }v_{k},k=1,2,...,N, \end{array}$$

where the prior distribution of θ is given by (2) and $x_{k}\in \;R^{n},y_{k}\in \;R^{m},w_{k}\in \;R^{n_{w}},v_{k}\in \;R^{m}$, $w_{k}∼\;N\left(0,I_{n_{w}}\right),$ and $v_{k}∼\;N\left(0,I_{m}\right)$. The variables $w_{0},...,w_{N-1},v_{1},...,v_{N}$ are mutually independent. The initial condition is zero. The solution of (29) with initial condition $x_{0}=0$ has the form

(31) $$x_{k}=\sum_{i=0}^{k-1}A_{\theta }^{k-i-1}B_{\theta }u_{i}+\sum_{i=0}^{k-1}A_{\theta }^{k-i-1}G_{\theta }w_{i}.$$

If we denote $X=\mathrm{col}(x_{1},...,x_{N})$, $Y=\mathrm{col}(y_{1},...,y_{N})$, $U=\mathrm{col}(u_{0},...,u_{N-1})$, $W=\mathrm{col}(w_{0},...,w_{N-1})$, and $V=\mathrm{col}(v_{1},...,v_{N})$, then (31) and (30) can be rewritten as

(32) $$\begin{array}{c} X=B_{\theta }U+G_{\theta }W,\;\mathrm{and} \end{array}$$

(33) $$\begin{array}{c} Y=C_{\theta }X+D_{\theta }V, \end{array}$$

where the matrices $B_{\theta }$, $G_{\theta }$, $C_{\theta }$, and $D_{\theta }$ follow forms (30) and (31). The variables *W* and *V* are independent, where $W∼N(0,I_{Nn_{w}})$ and $V∼N(0,I_{Nm})$. Substituting (32) into (33) we get Equation (1), where $F_{\theta }=C_{\theta }B_{\theta }$, $Z=C_{\theta }G_{\theta }W+D_{\theta }V$. The conditional density of *Z* has the form $p\left(Z|\theta \right)=N\left(Z,0,S_{\theta }\right)$, where the covariance matrix is given by

(34) $$S_{\theta }=D_{\theta }D_{\theta }^{T}+C_{\theta }G_{\theta }G_{\theta }^{T}C_{\theta }^{T}.$$

Hence, the results of Section 2 can be applied to the dynamical system (29) and (30).

## 4. Example

In some fault detection and isolation problems [3,4], there is a need to determine which of the known models of the process is the most adequate. It is, therefore, important to find a signal that emphasizes the differences between these various models. As an example of this type of problem, let us consider three stochastic continuous-time models:

(35) $$dx=\left(A_{\theta }x+B_{\theta }u\right)dt+G_{\theta }dw,$$

where θ∈{1,2,3}, $x\left(t\right)\in R^{\theta }$, x(0)=0, u(t),w(t)∈R, *w* is a standard Wiener process, and

(36) $$\begin{array}{c} A_{1}=-1,B_{1}=1,G_{1}=0.05, \end{array}$$

(37) $$\begin{array}{c} A_{2}=\left[\begin{array}{cc} 0 & 1\\ -3 & -2.5 \end{array}\right],B_{2}=\left[\begin{array}{c} 0\\ 3 \end{array}\right],G_{2}=\left[\begin{array}{c} 0\\ 0.05 \end{array}\right], \end{array}$$

(38) $$\begin{array}{c} A_{3}=\left[\begin{array}{ccc} 0 & 1 & 0\\ -3 & -3.5 & 1\\ 0 & 0 & -10 \end{array}\right],B_{3}=\left[\begin{array}{c} 0\\ 0\\ 30 \end{array}\right],G_{3}=\left[\begin{array}{c} 0\\ 0\\ 0.05 \end{array}\right]. \end{array}$$

**Figure 1:**
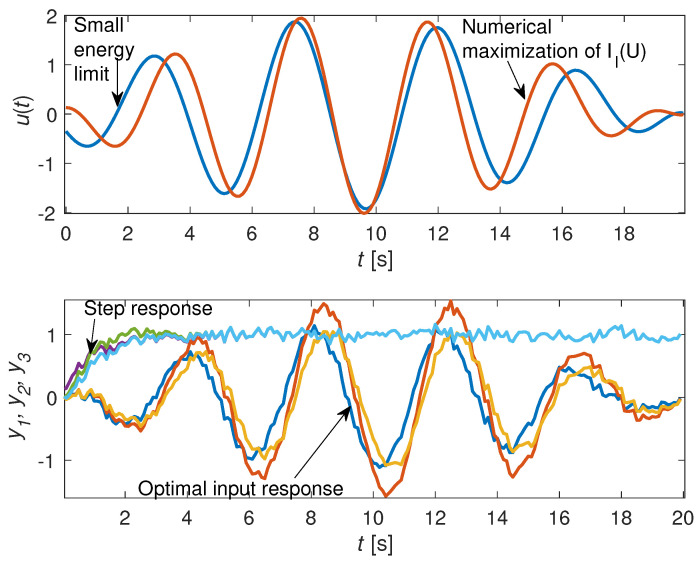
(**Top**) Numerical solution of (19) and the small energy approximation (26), for ϱ=N=200. (**Bottom**) Step responses and optimal responses of all systems.

The step responses of these models are similar and they are difficult to experimentally distinguish from each other if the noise level is significant. The observation equation has the form

(39) $$y_{k}=x_{1}\left(t_{k}\right)+0.05v_{k},k=1,2,...,N,$$

where $v_{k}∼N\left(0,1\right)$, $t_{k}=kT_{0}$, $T_{0}=0.1$, and $x_{1}$ is the first component of x(t). If $x_{k}=x\left(t_{k}\right)$ and $u\left(t\right)=u_{k},t\in \left[t_{k-1},t_{k}\right)$, then, after discretization, the state $x_{k}$ and the output $y_{k}$ are described by (29) and (30), with appropriate matrices $A_{\theta }$, $B_{\theta }$, $C_{\theta }$, $G_{\theta }$, and $D_{\theta }$. The matrices $F_{\theta }$ and $S_{\theta }$ are calculated by using (31)–(34). Let us observe that, although the orders of the systems are different, the size of both $F_{\theta }$ and $S_{\theta }$ is always N×N. We are interested in the maximization of $I_{l}\left(U\right)$. The solutions of (19) and (26) with a uniform prior, ϱ=N, and N=200 steps, are shown in the upper part of Figure 1. The step responses and the optimal responses are shown in the bottom part of Figure 1.

Let us observe that, in contrast to the step signal, the optimal signal clearly distinguishes the systems—although the energy of all input signals was the same and equal to *N*.

**Figure 2:**
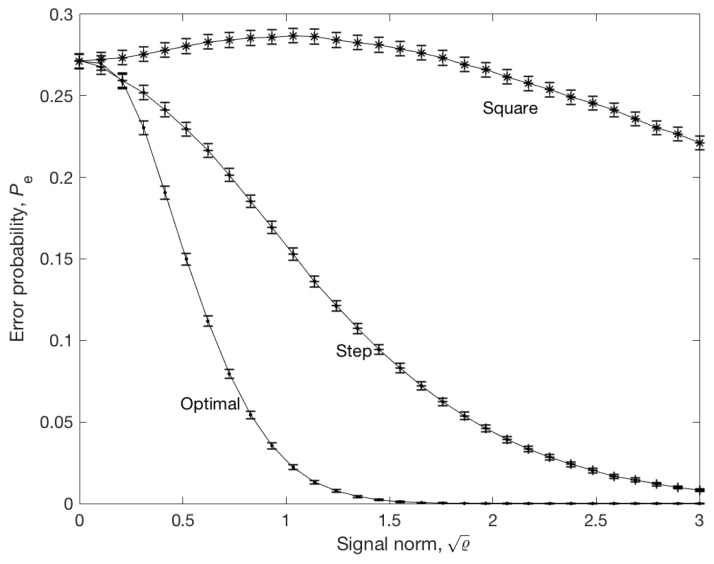
Error probability of the MAP estimator for the optimal signal (.), step signal (+), and square (*) signal with period of three. The number of steps is N=50. The error probability has been estimated by a Monte Carlo method with $10^{6}$ trials. Standard error bars were multiplied by factor of 10 for better visibility.

Let $U_{st},U_{sq}\in \partial S_{1}$ denote the normalized step and square signal with period of three, respectively, and let $U^{*}\left(\varrho \right)$ denote the optimal signal. To check the validity of the results, the error probabilities $P_{e}\left(\varrho U_{st}\right)$, $P_{e}\left(\varrho U_{sq}\right)$, and $P_{e}\left(U^{*}\left(\varrho \right)\right)$ were estimated by Monte Carlo simulation with $10^{6}$ trials and N=50 steps. The results are shown in Figure 2. It was observed that the optimal signal gives an error probability several thousand times smaller than the step or square signal with the same energy. The second observation is that $P_{e}\left(\varrho U_{sq}\right)$ initially increased with ϱ. To explain this, let us note that Inequality (17) does not guarantee that $P_{e}$ is decreasing function of ϱ. Hence, it is possible that $P_{e}$ increases in certain directions, although Theorem 2 guarantees that $P_{e}$ tends to zero, provided that signal norm tends to infinity.

## 5. Comparison with the Average D-Optimal Design

Classical methods of signal design for parameter identification use various functionals of the Fisher information matrix as a utility function. One of the most popular is D-optimal design, which consists of finding a signal that maximizes the determinant of the information matrix (see [10,11,12] and the review article [9]). These methods are well-suited to models that are linear in their parameters. Unfortunately, in typical identification and discrimination tasks, the output is a non-linear function of the parameters and the information matrix depends on unknown parameters to be identified. One of the possibilities for avoiding this problem is the averaging of the utility function over the prior parameter distribution. This method is called average D-optimal design (see [14,26] and [9] (Sections 5.3.5 and 6), for details). The Bayesian design, described in the previous sections, will be compared with the average D-optimal design. To that end, let us consider a finite family of linear models (see also [12] (pp. 91–93))

(40) $$y_{k}=\frac{b_{\theta }z^{-1}}{1-a_{\theta }z^{-1}}u_{k}+\sigma_{v}v_{k},$$

where θ∈{1,2,3,4}, $a_{\theta }=0.6+0.1\left(\theta -1\right)$, $b_{\theta }=1-a_{\theta }$, $\sigma_{v}=0.1$, and $v_{k}∼N\left(0,1\right)$. The prior distribution of θ is uniform (i.e., $p_{0,\theta }=0.25$). The state space representation of (40) has the form

(41) $$\begin{array}{c} x_{k+1}=a_{\theta }x_{k}+b_{\theta }u_{k}, \end{array}$$

(42) $$\begin{array}{c} y_{k}=x_{k}+\sigma_{v}v_{k}, \end{array}$$

which is consistent with (29) and (30). The Fisher information matrix is given by

(43) $$M_{F}\left(\theta ,U\right)=\frac{1}{N\sigma_{v}^{2}}\sum_{k=1}^{N}d_{k}d_{k}^{T},$$

where $d_{k}={\left(\xi_{k},\eta_{k}\right)}^{T}$ and $\xi_{k}=\frac{\partial y_{k}}{\partial a_{\theta }}$, $\eta_{k}=\frac{\partial y_{k}}{\partial b_{\theta }}$, denote the sensitivity of the output $y_{k}$ to changes in parameters *a* and *b*, respectively. The derivatives $\xi_{k}$ and $\eta_{k}$ fulfil the sensitivity equations

(44) $$\begin{array}{c} \xi_{k}=2a_{\theta }\xi_{k-1}-a_{\theta }^{2}\xi_{k-2}+b_{\theta }u_{k-2}, \end{array}$$

(45) $$\begin{array}{c} \eta_{k}=a_{\theta }\eta_{k-1}+u_{k-1},k=1,2,...,N, \end{array}$$

with zero initial conditions. The average D-optimal design consists in finding a signal *U* that maximizes the expectation of the determinant of the information matrix (see [9] (Sections 5.3.5 and 6), [11,14] (Chapter 6), and [12] for details). Hence, the utility function to be maximized has the form

(46) $$J\left(U\right)=\sum_{\theta =1}^{4}p_{0,\theta }\left|M_{F}\left(\theta ,U\right)\right|,$$

with the energy constraints given by (3). Maximization of the utility function (46) has been performed for various signal energies and the error probability of the MAP estimator was estimated by Monte Carlo with $10^{5}$ trials. The same procedure was repeated using Bayesian design for (41) and (42). The results are shown in Figure 3. The error rate of Bayesian method is significantly smaller when compared to D-optimal design, at least in this example. In particular, the signal shown in the upper-right part of Figure 3 gives an error probability approximately three times smaller than that of D-optimal signal, although the energy of both signals was the same.

## 6. Possible Extensions of the Results

In this section, we will briefly discuss some possible extensions of the results to an infinite set of parameters and beyond linear and Gaussian models.

### 6.1. Non-Linear Models

Although the article refers to linear models, it is possible to extend the results to non-linear models of the form

(47) $$Y=F_{\theta }\left(U\right)+Z,$$

where the conditional density of variable *Z* is given by

(48) $$p\left(Z|\theta ,U\right)=N(Z,0,S_{\theta }\left(U\right))$$

and $S_{\theta }\left(U\right)>0$, for all $U\in U_{ad}$, θ∈{1,...,r}. Under these assumptions, the density of *Y* still remains a Gaussian mixture and the information lower bound takes the form

(49) $$I_{l}\left(U\right)=-\sum_{i=1}^{r}p_{0,i}\ln\left(\sum_{j=1}^{r}p_{0,j}e^{-D_{i,j}\left(U\right)}\right),$$

where

(50) $$\begin{array}{c} D_{i,j}\left(U\right)=\frac{1}{4}{\left(F_{i}\left(U\right)-F_{j}\left(U\right)\right)}^{T}{\left(S_{i}\left(U\right)+S_{j}\left(U\right)\right)}^{-1}\left(F_{i}\left(U\right)-F_{j}\left(U\right)\right)+\\ +\frac{1}{2}\ln|\frac{1}{2}\left(S_{i}\left(U\right)+S_{j}\left(U\right)\right)|-\frac{1}{4}\ln|S_{i}\left(U\right)||S_{j}\left(U\right)|. \end{array}$$

### 6.2. Non-Gaussian Models

If p(Z|θ,U) is non-Gaussian distribution, then it is possible, on the basis of Equation (10) in [24], to construct an information lower bound of the form

(51) $$I_{l}\left(U\right)=-\sum_{i=1}^{r}p_{0,i}\ln\left(\sum_{j=1}^{r}p_{0,j}e^{-C_{\alpha }(p_{i}\left|\right|p_{j})}\right),$$

where

(52) $$C_{\alpha }(p_{i}\left|\right|p_{j})=-\ln\int p{\left(Z|i,U\right)}^{\alpha }p{\left(Z|j,U\right)}^{1-\alpha }dZ$$

is the Chernoff α-divergence and α∈[0,1]. Unfortunately, calculation of (52) is difficult if $n_{Y}$ is large.

### 6.3. Infinite Set of Parameters

Let us consider following model:

(53) Y=F(θ)U+Z,

where p(Z|θ)=N(Z,0,S(θ)) and $\theta \in R^{p}$. If we assume that prior density of θ is Gaussian; that is,

(54) $$p_{0}\left(\theta \right)=N\left(\theta ,m_{\theta },S_{\theta }\right),S_{\theta }>0,$$

then

(55) $$p\left(Y\right)=\int p_{0}\left(\theta \right)N\left(Y,F\left(\theta \right)U,S\left(\theta \right)\right),$$

and p(Y) can be approximated by a finite Gaussian mixture

(56) $$p\left(Y|U\right)=\int p_{0}\left(\theta \right)N\left(Y,F\left(\theta \right)U,S\left(\theta \right)\right)d\theta \approx \sum_{j=1}^{N_{a}}p_{0,j}N\left(Y,F\left(\theta_{j}\right)U,S\left(\theta_{j}\right)\right),$$

where $p_{0,j}⩾0$ and $\sum_{j=1}^{N_{a}}p_{0,j}=1$. It’s possible to calculate weights and nodes in (56) by using multidimensional quadrature. If $n_{\theta }$ is large, then an appropriate sparse grid should be used. To illustrate the method, we will show only a simple, second-order quadrature with $2n_{\theta }$ points.

**Lemma** **3.** *The approximate value of the integral $J\left(f\right)=\int N\left(\theta ,m_{\theta },S_{\theta }\right)f\left(\theta \right)d\theta$ is given by*

(57) $$J\left(f\right)\approx \frac{1}{2n_{\theta }}\sum_{j=1}^{2n_{\theta }}f\left(\theta_{j}\right),$$

*where*

(58) $$\theta_{2i-1}=m_{\theta }-S_{\theta }^{0.5}e_{i},\theta_{2i}=m_{\theta }+S_{\theta }^{0.5}e_{i},i=1,...,n_{\theta }$$

*and $e_{i}$ is $i^{th}$ basis vector. If $f\left(\theta \right)=\frac{1}{2}\theta^{T}A\theta +b^{T}\theta +c$, then equality holds in (57).*

**Proof.**  Direct calculation. □

Application of Lemma 3 to (56) gives $p_{0,j}=p_{0}={\left(2n_{\theta }\right)}^{-1}$, $N_{a}=2n_{\theta }$. Now, since (56) is a Gaussian mixture, the results of Section 2 can be utilized and the information lower bound takes the form

(59) $$I_{l}\left(U\right)=-p_{0}\sum_{i=1}^{r}\ln\left(\sum_{j=1}^{r}e^{-D_{i,j}\left(U\right)}\right)-\ln p_{0},$$

where $D_{i,j}$ and $\theta_{j}$ are given by (15), (16), and (58), respectively, and $F_{j}=F\left(\theta_{j}\right)$, $S_{j}=S\left(\theta_{j}\right)$. The approximate solution of (12) can be found by maximization of (59) with the constraints (3).

## 7. Discussion and Conclusions

An effective Bayesian design method for linear dynamical model discrimination has been developed. The discrimination task is formulated as an estimation problem, where the estimated parameter θ indexes particular models. To overcome the computational complexity, instead of I(Y;θ|U), its lower bound ($I_{l}\left(U\right)$), proposed by Kolchinsky & Tracey [24], was used as a utility function. This bound is especially useful, as it is differentiable, tight, and reaches the maximum available information H(θ). It has been proved, on the basis of the results of Feder & Merhav [20], that the error probability of the MAP estimator (see Lemma 2) is upper bounded by $\frac{1}{2}\left(H\left(\theta \right)-I_{l}\left(U\right)\right)\log_{2}e$. The maximization of $I_{l}\left(U\right)$ has been considered under the signal energy constraint, but other kinds of constraints can also be easily implemented. It was shown that the maximization of $I_{l}\left(U\right)$, in the case of two parameters, is equivalent to maximization of a quadratic form on the sphere (see also [3]). Next, the small energy limit was analyzed. It was proved that the solution is given by an eigenvector corresponding to maximal eigenvalue of some Hermitian matrix. This result can serve as starting point for numerical maximization of $I_{l}\left(U\right)$. If the energy of the signal tends to infinity, then almost all (in the sense of the Lebesgue measure) signals generate maximum information, provided that the impulse responses of the models are pairwise different. Under these conditions, it was proved that $P_{e}$ of the MAP estimator tends to zero.

An example of discrimination of three stochastic models with different structures was given. It is easy to observe, from Figure 1, that, in contrast to the step signal, the optimal signal clearly distinguished the systems, although the energy of both signals was the same. The $P_{e}$ of the MAP estimator was calculated by Monte Carlo simulation. It was observed that the square signal gave an error probability several thousand times greater than the optimal signal with the same energy. Hence, we conclude that the error probability and the accuracy of MAP estimator depends very strongly on the excitation signal. Although Theorem 2 implies that $\lim_{\varrho \to \infty }P_{e}\left(\varrho U\right)=0$ for almost all *U*, there exist signals such that $P_{e}\left(\varrho U\right)$ is locally increasing. This is the case of a high-frequency square signal, as illustrated in Figure 2.

It was shown, in Section 5 (see Figure 3), that $P_{e}$ of the MAP estimator corresponding to Bayesian design was a few times smaller than $P_{e}$ generated by D-optimal design, at least in the analyzed example. This result suggests that Bayesian design can be applied to non-linear problems and that it is superior to classical D-optimal design.

Some extensions of the results to the infinite set of parameters and beyond linear and Gaussian assumptions were briefly discussed in Section 6. Extension to non-linear models seems to be easy, but the non-Gaussian case is difficult and a deeper analysis is required. The case of an infinite set of parameters was discussed in Section 6.3. It was shown that the measurement density can be approximated by a finite Gaussian mixture, after which the results of Section 2 could be directly applied. The general conclusion of this analysis is that the information bounds can be easily constructed, as long as the measurement density is a mixture of Gaussian distributions.

An analytical gradient formula must be provided for the effective numerical maximization of $I_{l}\left(U\right)$. The matrix inversions and the determinants in (15) and (16) should be calculated by SVD. To reduce the computational complexity and required memory resources, the symmetries appearing in (15) and (16), and the fact that $D_{i,i}=0$, should be utilized. The determinants in (15) and the matrices $F_{i},S_{i}$ can be calculated off-line, but the matrices $Q_{i,j}$ may require too much memory if *N* and *r* are large. Therefore, $Q_{i,j}$ was calculated on-line.

Applications of the presented methods in dual control problems [5] are expected as part of our future work. An additional area in applications is the issue of automated testing of dynamical systems.

## Figures and Tables

**Figure 3:**
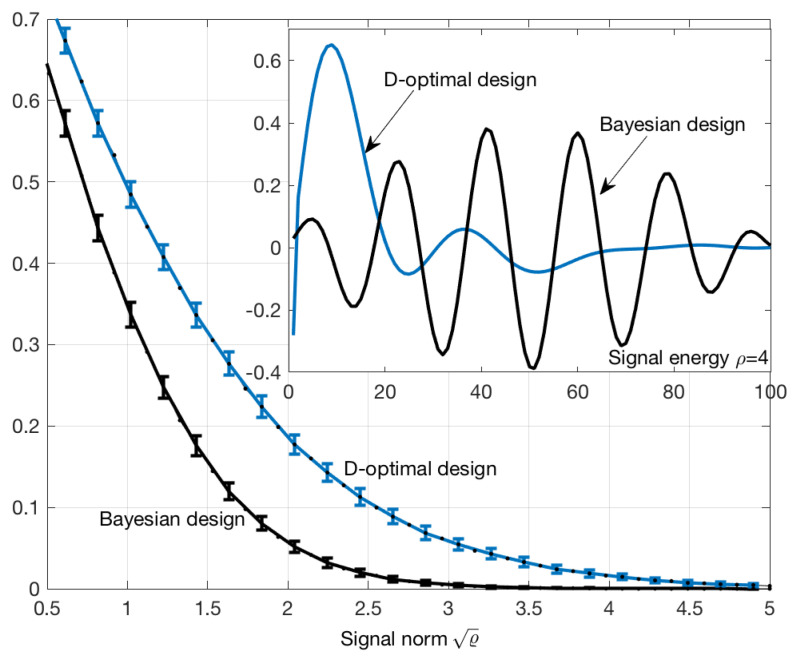
Error probability of theMAP estimator (see Section 2), as a function of signal norm and the exemplary input signals (top right) generated by D-optimal and Bayesian methods. The error probability was calculated by a Monte Carlo method with $10^{5}$ trials. Standard error bars were multiplied by a factor of 10 for better visibility.

## Abbreviations

ξ∼N(m,S) means that ξ has normal distribution with mean *m* and covariance *S*. The density of a normally-distributed variable is denoted by $N\left(x,m,S\right)=2\pi^{-\frac{n}{2}}{\left|S\right|}^{-\frac{1}{2}}\exp\left(-0.5{\left(x-m\right)}^{T}S^{-1}\left(x-m\right)\right)$. The symbol $\mathrm{col}(a_{1},a_{2},...,a_{n})$ denotes a column vector. The set of symmetric, positive-definite matrices of dimension *n* is denoted by $S^{+}\left(n\right)$.
